# Cerebral Fat Embolism Syndrome in a Patient With an Aortic Dissection and Orthopedic Injuries: A Case Report

**DOI:** 10.7759/cureus.34500

**Published:** 2023-02-01

**Authors:** Samuel Gearhart, Anthony Nguyen, Awais Z Vance

**Affiliations:** 1 Neurosurgery, Baylor Scott & White Medical Center - Temple, Temple, USA

**Keywords:** stroke, magnetic resonance imaging, trauma, neurosurgery, cerebral fat embolism syndrome

## Abstract

Traumatic brain injury is a significant cause of morbidity and mortality in adults and can be associated with severe secondary complications, including post-traumatic cerebral infarction. One potential cause of post-traumatic cerebral infarction is cerebral fat embolism syndrome (FES). We present a case in which a male in his twenties was involved in a motorcycle collision with a truck. He sustained numerous injuries, including bilateral femur fractures, left acetabular, open left tibial and fibular fractures, and a type A aortic dissection. Before orthopedic fixation, his Glasgow Coma Score (GCS) was 10. Following open reduction and internal fixation, his GCS was noted to be 4, with a stable computed tomography scan of the head. The differential included embolic strokes related to his dissection, an unrecognized cervical spine injury, and cerebral FES. Stat magnetic resonance imaging of the head demonstrated a starfield pattern of restricted diffusion consistent with cerebral FES. An intracranial pressure (ICP) monitor was placed, and his ICP acutely spiked to over 100 mmHg despite maximal medical management. This case highlights several key learning points, namely, that cerebral FES should remain in the mind of any physician treating high-energy multisystem traumas. While it is a rare syndrome, its effects can lead to significant morbidity and mortality as treatment is controversial and can conflict with the treatment of other systemic injuries. Further research into prevention and treatment is warranted to continue optimizing outcomes following cerebral FES.

## Introduction

Traumatic brain injury (TBI) is a significant cause of adult morbidity and mortality and stands as the leading cause of death in the first 45 years of life [1]. There are numerous secondary sequelae after TBI, one of the most severe being post-traumatic cerebral infarction, a serious complication with mortality of up to 75% [2]. There are likely multiple etiologies of post-traumatic stroke, including cerebral vasospasm, high intracranial pressure (ICP), cerebral hypoperfusion, blunt cerebrovascular injury, focal mass effects from hematoma, thromboembolic stroke, and in instances of multisystem trauma, fat embolism [3]. Here, we present the case of a patient with a TBI and multisystem traumatic injuries who developed neurological decline after orthopedic fixation. The etiology of his decline was not immediately evident due to his multitude of injuries, and his workup represented a diagnostic conundrum.

## Case presentation

**Figure 1:**
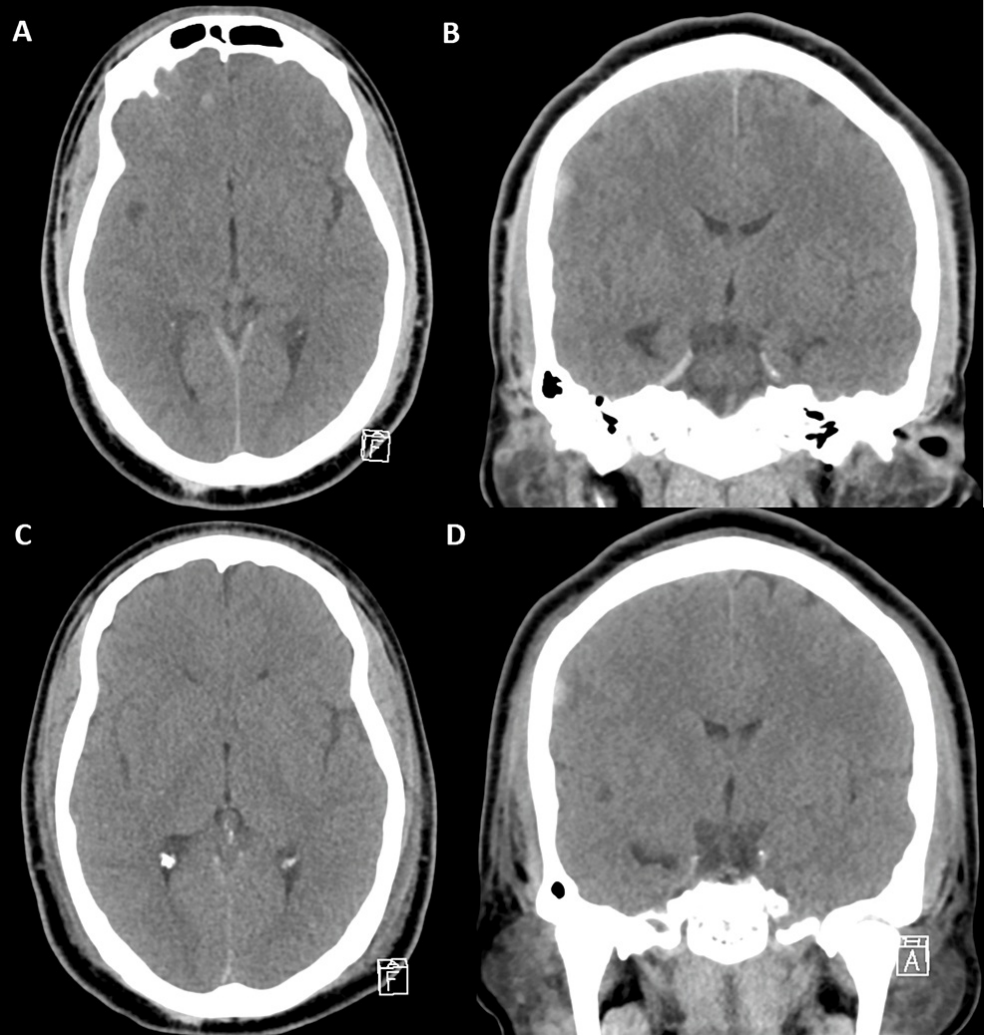
Axial and coronal slices of computed tomography scans of the head demonstrating a small right convexity subdural hematoma without herniation before orthopedic fixation of fractures (A and B), which remained stable postoperatively (C and D).

A male in his twenties collided with a truck while riding his motorcycle and was brought to an outside hospital. On presentation to the outside emergency department, he had a Glasgow Coma Score (GCS) of 3 and was intubated. He was found to have numerous orthopedic injuries, including bilateral femur fractures, a left acetabular fracture, open left tibia and fibula fractures, a left-sided hemothorax, type A aortic dissection, pericardial effusion, and left external auditory canal and mastoid fractures. He required a large-volume transfusion for severe hypotension. He was temporarily stabilized, and three hours after injury, he was transferred to our facility, where computed tomography (CT) scan of the head demonstrated a small volume of scattered traumatic subarachnoid hemorrhage, bifrontal intraparenchymal hemorrhages, and a small volume of right convexity subdural hematoma (Figures 1A-1B). Sedation was paused, and on examination, he awakened to voice, followed commands in his bilateral upper extremities, and had spontaneous antigravity movement of all extremities. He was assigned a GCS of 10T. Sedation was restarted, as he quickly became agitated and thrashed about despite his numerous fractures. The Orthopedic Surgery team took him to the OR for fixation of his femoral and tibial fractures. A head CT scan was obtained immediately postoperatively to evaluate the stability of his intracranial injuries. The CT scan did not demonstrate any adverse changes such as significant progression of prior findings, large-vessel infarction, or hydrocephalus (Figures 1C-1D). He was returned to the intensive care unit and intubated.

**Figure 2:**
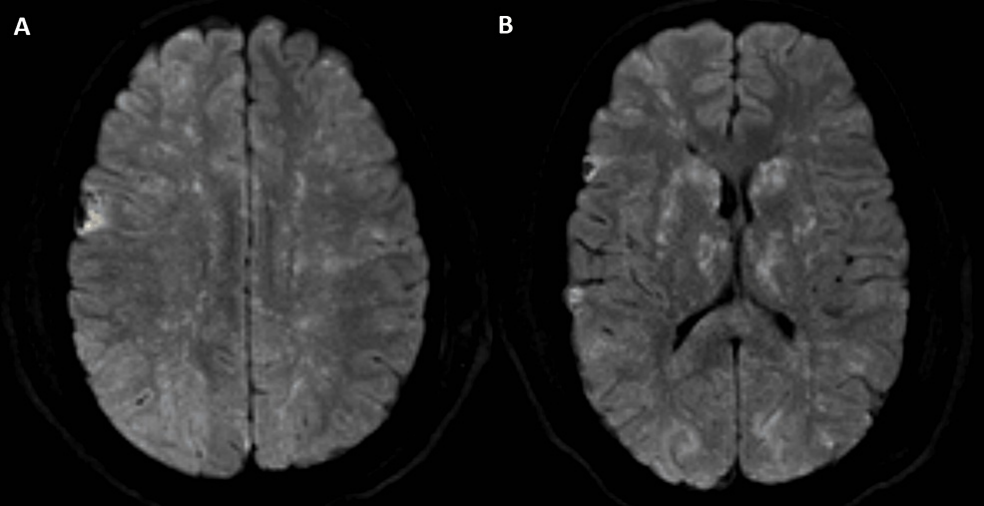
Axial slices of magnetic resonance imaging of the head with diffusion-weighted imaging demonstrating a diffuse starfield pattern of diffusion restriction, a hallmark finding of cerebral fat embolism syndrome.

Upon postoperative neurological examination, his GCS was 4 (subscores 2/1/1): noxious stimuli elicited eye movement without any extremity motor response. His pupils were equal, round, and reactive to light. He did not track movement with his eyes. Corneal, cough, and gag reflexes were present. Given the significant change in his neurologic exam with negative changes on the CT head, there was a concern for new neurological injury, and differential diagnoses included cervical spinal injury, diffuse axonal injury (DAI) with associated cerebral edema, or embolic showering from his aortic injury. Stat magnetic resonance imaging (MRI) of the cervical spine and brain was obtained. The MRI of the cervical spine was unrevealing, but the MRI of the brain demonstrated diffuse tiny foci of restricted diffusion in a starfield pattern (Figure 2). Given his multiple long bone fractures, this finding was most consistent with cerebral fat embolism syndrome (FES).

Our treatment then focused on managing ICP. An ICP monitor was placed, and tiered therapy was begun with hyperventilation, hypertonic saline, and mannitol. The patient’s ICP remained refractory to maximal medical management, and within three hours of placement of monitor placement, his ICP increased to over 100 mmHg and he acutely herniated. After a discussion with his family and later brain death testing, his family decided to proceed with organ donation.

## Discussion

This case is of educational value and illustrates several key learning points. The multisystem effects of high-energy trauma resulted in TBI, an aortic dissection, and multiple orthopedic injuries. As such, the initial differential for the acute change in his postoperative exam was quite broad, including worsening DAI, thromboembolism from his dissection, an unrecognized unstable cervical spine injury, and cerebral fat embolism. Blossoming of his DAI and intracranial hemorrhages was of primary concern in the immediate postoperative period, but a stable-appearing postoperative CT head ruled out worsening intracranial hemorrhage and edema. His cervical spine was unremarkable on MRI. Post-traumatic cerebral infarction was next in our differential diagnosis, but the mechanism behind the infarct remained in question. There was a concern for cerebral ischemia due to intraoperative hypotension or intracranial hypoperfusion, but there were no known instances of sustained intraoperative hypotension and no documentation of large-volume blood loss. Finally, there was a concern for a thromboembolic stroke given his aortic dissection. His MRI brain revealed a *starfield pattern* of diffusion restriction consistent with FES.

FES is a clinical syndrome seen in less than 0.1% of trauma patients, most often in those with long bone or pelvic fractures. The syndrome is most commonly seen in young adult males with femur fractures. Symptoms are most often seen between 24 and 48 hours after trauma, although patients with a patent foramen ovale may have a more acute presentation of cerebral FES [4,5]. The incidence of FES has been shown in some studies to increase with the number of fractures sustained [6]. Similarly, the correlation between the number and type of fractures and multisystem injury has been demonstrated in several studies, as traumatic mechanisms with high enough energy to cause multiple orthopedic injuries would logically have the potential to cause multisystem injury [7,8]. The management of potential FES in these cases may cause conflicting intersystem treatment plans. While there is no specific therapy for FES, steroids and, more controversially, heparin infusions have sometimes been advocated. In patients with TBI with or without an intracranial hemorrhage, the treatment of FES and TBI may oppose one another. This can pose a significant challenge in the care of the patient. Consequently, the possibility of FES should remain in the physician's mind when caring for polytrauma patients.

While the condition presently has a poor mortality rate with no definitive treatment, an interesting possibility in the management of polytrauma patients may lie in inferior vena cava (IVC) filter placement. Although few, some studies have sought to establish the effect of prophylactic IVC placement in polytrauma patients with long bone fractures. A study by Elkbuli et al. suggested that while prophylactic IVC filter placement was associated with higher rates of deep venous thrombosis and nonfatal pulmonary embolism in trauma patients, there was a notable association with lower in-hospital mortality compared to venous thromboembolism chemoprophylaxis alone [9]. This suggestion has been demonstrated in a case report by Di Bari et al. [10], who reported the prevention of recurrent FES in a 16-year-old with fractures of the femur and tibia by placing a prophylactic IVC filter. The usage of IVC filters for prophylaxis against cerebral fat embolism could significantly change patient care and certainly merits further investigation.

## Conclusions

Cerebral FES remains a clinically challenging syndrome with neurosurgical relevancy due to its significant morbidity and mortality in multisystem trauma patients. Major clinical hurdles include elevated ICP and treatment modalities that are relatively contraindicated in young TBI patients. Our case involves a prime example of the demographic in whom suspicion for FES is most warranted - a young adult male with multiple lower limb fractures and multisystem injury who unfortunately herniated due to cerebral FES. Knowledge of the population at risk, injury patterns seen in the condition, and presentation of the patient are important for detecting these potential complications during interdisciplinary care.

## References

[1] Popescu C, Anghelescu A, Daia C, Onose G (2015). Actual data on epidemiological evolution and prevention endeavours regarding traumatic brain injury. J Med Life.

[2] Tawil I, Stein DM, Mirvis SE, Scalea TM (2008). Posttraumatic cerebral infarction: incidence, outcome, and risk factors. J Trauma.

[3] Harrigan MR (2020). Ischemic stroke due to blunt traumatic cerebrovascular injury. Stroke.

[4] Vetrugno L, Bignami E, Deana C (2021). Cerebral fat embolism after traumatic bone fractures: a structured literature review and analysis of published case reports. Scand J Trauma Resusc Emerg Med.

[5] Kainoh T, Iriyama H, Komori A, Saitoh D, Naito T, Abe T (2021). Risk factors of fat embolism syndrome after trauma: a nested case-control study with the use of a nationwide trauma registry in Japan. Chest.

[6] Gupta A, Reilly CS (2007). Fat embolism. Contin Educ Anaesth Crit Care Pain.

[7] Park S (2012). Clinical analysis for the correlation of intra-abdominal organ injury in the patients with rib fracture. Korean J Thorac Cardiovasc Surg.

[8] Mirza A, Ellis T (2004). Initial management of pelvic and femoral fractures in the multiply injured patient. Crit Care Clinics.

[9] Elkbuli A, Ehrhardt JD Jr, Kinslow K, McKenney M (2021). Prophylactic inferior vena cava filters: outcomes in severely injured trauma patients. Am Surg.

[10] Di Bari S, Bisulli M, Russo E (2021). Preoperative vena cava filter placement in recurrent cerebral fat embolism following traumatic multiple fractures. Scand J Trauma Resusc Emerg Med.

